# Supplementary material for: Relationship between health literacy and health-related quality of life in Korean adults with chronic diseases
Source: PLoS One. 2024 Apr 18;19(4):e0301894. doi: 10.1371/journal.pone.0301894 (PMC11025905; doi:10.1371/journal.pone.0301894)
Supplement: Attachment — Submitted filename: Rebuttal.docx [file pone.0301894.s001.docx]

I really appreciate the time and effort that the editor and the reviewer took to review my manuscript and give such detailed and thoughtful comments. I tried to address all comments as best as I could. However, if something falls short, please let me know.

Reviewer #1: Dear author,

Many thanks for the exciting manuscript. The presentation is largely solid. Below are a few points that could supplement the manuscript.
In general, the work of the Asian Health Literacy Association (AHLA), of which South Korea is also a member, could be discussed in a few sentences. This would be of interest to the international readership. For articles on national aspects of health literacy, it would be helpful to add national circumstances (education system, socioeconomics, utilisation of the healthcare system). This does not have to be described in detail. But appropriate sources would be helpful.
🡪 Thank you for positive feedback. Please see below.

Introduction
The work of the AHLA could be mentioned in the introduction. In addition, something should be mentioned about the Korean education system in the context of health literacy so that the international readership can categorise it.
Otherwise, the points listed above would be useful in the introduction.
🡪 I mentioned the work of the AHLA, Korean education system, health care system briefly in the introduction. [lines 52-56]

Methods:
Justify the use of HLS-EU 16! At the time of the study, the HLS-SF12 had already been successfully validated in Asia [Duong et al., 2019].
🡪 I could not identify the published rationale for why the Korea Health Panel Survey used HLS-EU 16 instead of HLS-EU 12. However, based on my conjecture, I tried to justify the use of HLS-EU 16 and added the reference [Duong et al., 2019]. [lines 135-138]

The choice of covariates should be justified. Most of them are plausible. However, for example, the severity of an illness rather than the number of illnesses seems to me to be more important. Or number of household members seems more logical to me than marital status. Please explain the choice of covariates.
🡪 This study is based on self-report data in a nationwide survey so that collecting severity of an illness would have been difficult if not impossible. I chose to include marital status because it is a sociodemographic variable that is shown to be associated with EQ-5D. I also added a reference [Han K-T, Kim SJ, Song H, Chun S-Y, Kim CO, Kim J-S, et al. Associations between quality of life and marital status in cancer patients and survivors. Asian Pac J Cancer Prev. 2014;15: 5287–5291. doi:10.7314/apjcp.2014.15.13.5287]

When carrying out the logistic regression analysis, the question arises as to why binomial logistic regression was not used for dichotomous (binary) dependent variables. Please explain! In many places, the binomial variant would have been more meaningful.
🡪 I used binomial logistic regression.

Results
The results are presented in a solid table. However, the description of the results is rather brief. A more detailed description in terms of striking or special results would be useful. Especially those that are to be discussed.
With regard to the hypotheses mentioned in the introduction, the presentation of the results should also be expanded in this respect.
🡪 I added a paragraph at the end of the result section to reiterate key findings and highlight what is to be discussed. [lines 228-232]

Discussion
The discussion is sound and the limitations are clearly stated. However, the concept of hypothesis could also be included here.
🡪 I added phrases indicating that the results confirm the hypothesis. [lines 244-245]

Conclusion
Conclusion can be drawn!
🡪 I stated the conclusions in relation to the hypotheses again. [lines 304-305]

see method:
Duong TV, Aringazina A, Kayupova G, Nurjanah, Pham TV, Pham KM, Truong TQ, Nguyen KT, Oo WM, Su TT, Majid HA, Sørensen K, Lin IF, Chang Y, Yang SH, Chang PWS. Development and Validation of a New Short-Form Health Literacy Instrument (HLS-SF12) for the General Public in Six Asian Countries. Health Lit Res Pract. 2019 Apr 10;3(2):e91-e102. doi: 10.3928/24748307-20190225-01.]

As it is very difficult to get reviewers, I have performed one of the two reviews myself. I am reviewer 2. Except for two comments, I also agree with the comments of reviewer 1. One comment with which I do not agree is the first reviewer’s comment regarding binomial logistic regression. As far as I can see you have applied this kind of regression in exactly those cases in which it is necessary. A further comment with which I not completely agree is given in the first paragraph regarding the results. In contrast to reviewer 1, I do not think that the description is too brief. However, I share with reviewer 1 a feeling of not being completely satisfied with this description. My problem is caused by the fact that you mainly repeat the information given in the tables. I would like to have a description that focusses more on the more essential aspects in the data, i.e., that you already elaborate and highlight what is especially important. This might be similar with what reviewer 1 meant with: ‘A more detailed description in terms of striking or special results would be useful. Especially those that are to be discussed.’

I must also give two comments that I forgot in my review. The first comment regards the fact that you use the Rao-Scott chi-square test for testing differences between categorial variables. The usual approach is applying that chi-square test that has originally been presented by Pearson and that is presented in textbooks as the classical approach for analyzing cross-tables. Please, explain what the Rao-Scott chi-square test distinguishes from the Pearson chi-square and why you think that choosing this statistic is more appropriate for your problem. If there is no special reason for choosing the Rao-Scott chi-square test I would like you to rerun the corresponding analyses with Pearson’s chi-square. This statistic has the simplest and clearest meaning. The second additional comment regards the footnote of table 3. This footnote is: ‘p-values were obtained by using the Rao-Scott chi-square test and the ANOVA.’ This makes no sense because you report only one p-value per test and an ANOVA would not be appropriate in this context. Please report the one and only statistical procedure you have actually applied.

- When I checked the SAS output, there is no difference between the result of Pearson and that of Rao-Scott. However, the literature suggests that use of the standard Pearson chi-squared test is inappropriate for analysis of complex survey data due to correlation among units within the same cluster. I added a reference in the method section to justify the use of Rao-Scott. [lines 173-174]
- Sorry, that was a confusing footnote. I clarified the footnote as follows “p-values for EQ-5D dimensions were obtained by using the Rao-Scott chi-square test and the p-value for mean EQ-5D index score by the ANOVA.” [line 213]

Reviewer #2: Review regarding the manuscript ‘Relationship between health literacy and health-related quality of life in Korean adults with chronic diseases’

A study regarding the relationship between health literacy and health-related quality of life in Korean adults with chronic diseases is presented in the manuscript. The study is performed with cross-sectional data taken from the Korea Health Panel Survey from 2021. The topic of the manuscript is interesting. The analyses are methodologically sound, and the different consideration are well ordered. However, there are several minor flaws. Most of them refer to the language. I have tried to address all of these flaws, including those regarding the language. However, I am not a native speaker of the English language. Therefore, I recommend having the manuscript being checked by a native speaker. Should this person make different propositions than I make, please discuss this with the native speaker and choose that solution that the native speaker and you finally prefer. In the following text, you find the numerous minor flaws that should be corrected before publication.
🡪 Thank you again for having gone such a length to improve my manuscript. I tried to address all your comments.

Lines 21-22
‘This aims to examine the relationship between health literacy and HRQoL among Korean adults with chronic conditions. This cross-sectional study was based on the Korea Health Panel Survey in 2021.’
I suggest replacing this by
‘In this contribution, the relationship between health literacy and HRQoL in Korean adults with chronic conditions is examined using data of the cross-sectional Korea Health Panel Survey from 2021.’
🡪 revised as recommended. [lines 22-24]

Line 25 and further text
I suggest replacing
‘EQ-5D index score’
by
‘EQ-5D index’
An index is a score. Therefore, ‘EQ-5D index score’ is one word too much.
🡪 revised as recommended. [throughout the manuscript]

Lines 26 to 28
‘Multiple logistic regression models were implemented for domain-specific outcomes. In the weighted sample, 30.8%, 24.6%, and 44.6% had inadequate, marginal, and adequate levels of health literacy, respectively.’
A reader who has not read your manuscript cannot know what you meant by ‘domain-specific’ and, even a reader who has read you manuscript does not know what you mean by weighted by ‘weighted’? (See below)
Please revise the corresponding parts of the main text according to my suggestions and revise the abstract correspondingly.
🡪 I revised the main text and abstract, as per your recommendations. [throughout the manuscript]

Lines 28 to 30
‘EQ-5D index scores were positively associated with marginal (B=0.018, p<0.001) and adequate literacy (B=0.017, p<0.001) compared to inadequate literacy.’
I suggest writing
‘The EQ-5D index increases with marginal (B=0.018, p<0.001) and adequate literacy (B=0.017, p<0.001) compared to inadequate literacy.’
🡪 Revised as recommended [lines 29-30]

Lines 34 to 35
‘Inadequate health literacy is prevalent among Korean adults with chronic diseases.’
Should be reported earlier.
I intended to write this as a conclusion which was then placed near the end of the abstract. To improve the flow of sentences, I added the phrase, “In conclusion”. [lines 35-36]

*****

Lines 47 to 48
‘Nearly a half of adults in the U.S. have limited health literacy according a systematic review [6].’
I suggest replacing this by
‘According to a systematic review [6], nearly a half of adults in the U.S. have limited health literacy.’
🡪 Revised as recommended. [lines 49-50]

Line 48
I suggest replacing ‘meaning’ by ‘which means that’.
🡪 Revised as recommended. [line 50]

Lines 49 to 51
I suggest replacing
‘Nearly one in two adults aged 19 years and over in Korea have limited health literacy to make informed decision regarding their own health [8].’
By
‘In Korea, nearly one in two adults aged 19 years and over has limited health [8].’ The term ‘to make informed decision regarding their own health’ is not helpful in this sentence. The remaining changes regard the language.’
🡪 Revised as recommended. [lines 51-52]

Line 57
Please replace ‘component to effective treatment’ by ‘component of effective treatment’.
🡪 Revised as recommended. [line 74]

Line 59
Please replace ‘negatively can’ by ‘can negatively’.
🡪 Revised as recommended. [line 76]

Line 65
With regard to the language, you should replace ‘Reflective of’ by ‘Reflecting’ or ‘Being reflective of’. However, there is still a further problem in the sentence. HRQoL-indices as the EQ-5D-index do not reflect the individual experience. They reflect societal values.

🡪 Revised as recommended. While I agree that QoL reflects societal values, I wanted to mean the subjective experience in the sense that HRQoL is a patient-reported outcome as opposed to health outcomes diagnosed and reported by the health professional. [lines 82-83]

Line 65 to 77
Please report findings in present tense. They are usually meant to be generalizable over time.
🡪 I rewrote the paragraph in present tense. [lines 82-94]

Line 70 to 71
Replace ‘Type 2 diabetes is a chronic condition that garnered particular attention in relation to health literacy [22–24]’ by ‘A chronic condition that garnered particular attention in relation to health literacy is type 2 diabetes [22–24]’.
🡪 Revised as recommended. [lines 87-88]

Line 77
Please replace ‘Also’ by ‘Moreover’ or ‘Further’ or ‘Furthermore’.
🡪 Revised as recommended. [line 93]

Line 78
Please omit ‘using survey data based on a nationally representative large sample of the population’. Inserting this phrase makes the sentence very difficult to understand. Please give this information in the first sentence of the methods part.

🡪 Revised as recommended. [lines 95, 104-106]

Lines 83 and 84
Please formulate the hypotheses in present tense (see above).
🡪 Revised as recommended. [lines 99-100]

Line 91
‘city vs. province’
Why ‘vs’? I cannot be certain about what you want to say, but I guess the formulation ‘or, respectively,’ accords more to what you might want to express.

🡪 I used ‘or’ instead of ‘vs’ [line 108]

Line 92
The word ‘dong’ belongs to American slang and means, with absolute certainty, something that you do not want to talk about in this manuscript. The word ‘eup’ is not an English word at all. Please use English words. Moreover, my comment regarding ‘vs’ also applies to this line.
🡪 I could not come up with a better word to replace the Korean words (dong and eup). I thought about using ‘county’. However, county in the U.S. is a much bigger size than dong or eup. So I deleted the words altogether, which would not harm the meaning of the sentence. [line 108]

Line 95
I would leave out ‘presenting a rare opportunity to examine the hypotheses posited above’.
🡪 I deleted the phrase.

Line 111 to 119
I am not certain how this paragraph is meant. Is it a list of different health literacy measurement instruments included in the survey or is it consideration of the development of these measurement over time? If the first is true, you should make this clear. For example, you could write ‘KHPS contained several questionnaires addressing health literacy. These questionnaires are….’. If the second is true, this part belongs into the introduction.
🡪 It meant the latter. So I moved the paragraph to the introduction section. [lines 57-65]

Line 120 to 128
Please give first the general description of the HLS-EU-Q16 and then the report of the cultural adaptation.
🡪 I changed the order as recommended. [lines 127-135]

Lines 131 to 142
You should report that each dimension of the EQ-5D is addressed by exactly one item, and you should replace the formulation ‘domain specific responses’ by the formulation ‘responses to the individual EQ-5D items’ throughout the complete manuscript. This will make your presentation easier to understand.
🡪 Revised as recommended. [line 147]

Sub-chapter ‘Covariates’
Please, tell the reader purpose for which you want to control for confounding variables and why you believe that the variables you have included as possible confounders serve this purpose.
🡪 I added “These variables could potentially influence EQ-5D and therefore were adjusted for in all regression models to better estimate the relationship between health literacy and EQ-5D.” [lines 159-161]

Line 149
'…classified as ≤primary, middle school, high school, and ≥college’
What do the symbols ‘≤’ and ‘≥’ mean in this context? I guess that they should be omitted.
🡪 I revised the concerned part in the text and the tables into “no or primary education, middle school, high school, and college or higher” [line 161]

Line 150
Please replace ‘ever married’ by ‘formerly been married’.
🡪 I replaced ‘ever married’ by ‘formerly married.’ [line 163]

Line 151
I suggest using the denominations ‘employed’, ‘self-employed’ and ‘un-employed’ for categorizing employment status.
🡪 Revised as recommended. [line 163]

Sub-chapter ‘Statistical analysis’
Past-tense sounds better and is more often used in the analysis sub-chapter.
🡪 I made changes so that most sentences in statistical analysis were in past tense. [lines 170-190]

Line 164
As far as I can infer from your text, you actually apply multivariate and not multivariable regression. As far as I know, multivariable regression is a regression with more than one dependent variable.
🡪 I understand that these two terms are confusing. The literature guides us that multivariable regression is used for analysis with one dependent variable, which is one that I used.

References:

https://www.ncbi.nlm.nih.gov/pmc/articles/PMC3518362/ https://academic.oup.com/ntr/article/23/8/1446/5812038

Lines 169 to 170
‘All analyses adjusted for sampling weights that are used in the complex sampling design of the 2021 KHPS.’
I have no idea what is done here. Please explain in the text. What is weighted by what? To which purpose is this weighting performed? How is the weighting integrated in the analyses?
🡪 I added the following for better explanation; “The complex survey uses oversampling of some subgroups of the population and poststratification adjustments for nonresponses, which cause the issues of unequal selection probability across subgroups and incorrect estimation of standard errors [ref]. Sampling weights provided in complex survey data represent the inverse of selection probability. Units oversampled have a smaller weight value. Weighting was performed using the “surveyreg” and “surveylogistic” procedures in SAS. [lines 184-189]

Chapter ‘results’
I would use present tense throughout this chapter.
🡪 Revised as recommended.

Line 175
What do you want to say with ‘In all’ in this context?
Please replace ‘received’ by ‘had’.
🡪 I deleted ‘in all” and replaced ‘received’ by ‘have’, in keeping with your recommendation of using present tense for results. [line 195]

Line 182
Please place the adverb after the verb.
🡪 Revised as recommended. [line 202]

Chapter ‘Discussion’
I also would use present tense in this chapter. Moreover, this chapter is very long detailed. Most readers would be thankful if you shortened the discussion to a half of the present length and focus on the more general aspects.
🡪 I rewrote discussion in present tense. I tried to shorten the discussion as much as I could and got to slightly less than one third of its original size.

Line 219
Please replace ‘this present study’ by ‘the study presented here’.
🡪 Revised as recommended. [line 242]

Lines 220 to 222
‘In particular, multi-morbidity appears to be an influential factor to the prevalence of inadequate health literacy, which increased in patients with a greater number of chronic conditions.’
You have cross-sectional data. Therefore, you cannot infer from your data whether a relationship between two variables results from one variable influencing the other, neither can you, in case of an influence, infer from the data, which of the two variables is the cause and which the effect. Youd mention something like this later as limitation. Accordingly, you should be cautious with using words as influence in this context. Of course, you can speculate about causal relationships as far as additional knowledge regarding the object of investigating allows for. However, given my knowledge regarding the object of investigation I see only a few mechanisms in which multi-morbidity could influence health literacy. To be specific, I do think that dementia reduces health literacy. However, I actually assume that health literacy influences multi-morbidity. To be specific, the higher the health literacy is the more people behave in a health-promoting way and, consequently, the less people suffer from multi-morbidity. In the further text, you develop similar thoughts. You should make the text in lines 220 to 222 consistent with this line of thinking.
🡪 I completely agree. I deleted the controvertible part as it is not helpful in my thesis and to shorten the manuscript, which was recommended above.

Lines 238 to 240
‘This may be in part because low literacy adversely influenced adult patients’ ability to correctly interpret warning labels for prescription drugs [40].’
I suggest
‘This may partly be caused by low literacy adversely influencing adult patients’ ability to correctly interpret warning labels for prescription drugs [40].’
🡪 Revised as recommended. [lines 256-257]

Lines 252 to 254
‘However, after adjustments for covariates, adequate and marginal health literacy were associated with the odds of reporting no problems with self-care, mobility, and usual activities only.’
I would restrict to the category of adequate health literacy and, accordingly, include anxiety/depression into the set of dimensions associated with health literacy.
🡪 Revised as recommended. [lines 266-267]

Line 254
If you follow my suggestion to replace ‘domain specific responses’ by the formulation ‘responses to the individual EQ-5D items’ then you should replace in line 254 ‘domains’ by ‘dimensions’.
🡪 Revised as recommended. [line 268]

Chapter ‘Conclusion’ please also in present tense.

🡪 I rewrote conclusion in present tense.
